# Benign and Malignant Renal Cells Are Differentially Inhibited during Prolonged Organ Preservation

**DOI:** 10.1371/journal.pone.0081745

**Published:** 2013-12-09

**Authors:** Nengwang Yu, Shuai Fu, Yibao Liu, Zhihou Fu, Jianzhong Meng, Zhonghua Xu, Baocheng Wang, Aimin Zhang

**Affiliations:** 1 Urology Department, General Hospital of Jinan Military Command, Jinan, Shandong, China; 2 Department of Thoracic Medical Oncology, Beijing Cancer Hospital, Beijing, China; 3 Orthopedics Department, General Hospital of Jinan Military Command, Jinan, Shandong, China; 4 Blood Purification Department, General Hospital of Jinan Military Command, Jinan, Shandong, China; 5 Urology Department, Qilu Hospital, Jinan, Shandong, China; 6 Oncology Department, General Hospital of Jinan Military Command, Jinan, Shandong, China; UNIFESP Federal University of São Paulo, Brazil

## Abstract

The worry of potential residual renal cancer cells in donor kidney after resection of small renal cancer impedes the extensive use of such controversial donor source. To explore the impacts of organ preservation process on the survival of renal cancer cells, we detected cell proliferation and viability of benign and malignant renal cell lines and clinical renal samples after treated with simulated organ preservation process. It was found that the viability and proliferation of malignant renal cells are inhibited much more than that of benign renal cells during prolonged organ preservation. The inhibition of proliferation in benign renal cells is fully reversible, while in malignant renal cancer cells is not fully reversible after a certain time. So potential residual renal cancer cells could be partly inhibited and eliminated by organ preservation process.

## Introduction

Although cancer is generally not considered a transmissible disease, direct transmission from one host to another has been documented in rare circumstances. One such example arises from the immunosuppressive condition following transplantation of a donor organ harboring occult malignancy. Donor-transmitted cancers emerged in a relatively large numbers in the pioneering era of transplantation, since the risk was not well-recognized [1]. The transplant community has since learned that most cancers, including renal cancer, serve as contraindications for organ donation [2].

For patients with end-stage renal failure (ESRF), renal transplantation confers improvement in quality of life and survival when compared to dialysis. However, in the current era of scarce donor organs, a significant number of ESRF patients, especially those with severe medical problems on long-term dialysis, die from the complications of chronic renal insufficiency before they are able to receive a transplant [3]. Various measures, including the use of marginal donors, have been utilized to increase the donor pool. Small renal cancers, usually less than 4 cm in diameter, have low malignant potential; therefore, several transplant centers have explored the utilization of donor kidney after resection of small renal cancer [4]–[15].

It has been reported that 5.3% of small renal cancers are multifocal, and only a small part of these can be detected by routine image examination [16]. Therefore, it is possible that some residual renal cancer cells are present in these donor kidneys after resection of detectable malignant lesions, especially for those kidneys with endophytic or multifocal microcarcinomas. Before kidneys are transplanted, they are routinely subjected to the process of organ preservation. The most common kidney preservation process is perfusion with and cold-storage (at 4°C) in UW (University of Wisconsin) solution for several hours. The effects of this organ preservation process on the survival of residual renal cancer cells remain unclear, and may impact donor-transmitted renal cancer. To the best of our knowledge, no such studies have been conducted to address this question. Therefore, we conducted a preliminary study to explore the impact of this organ preservation process on the survival of renal cancer cells. Our results show that benign and malignant renal cells are differentially inhibited during prolonged organ preservation.

## Materials and Methods

### Cell culture

The human renal carcinoma cell line, 7860, and the human proximal tubule epithelial cell line HK-2 were purchased from the Chinese Academy of Sciences Cell Bank. HK-2 cells were maintained in Dulbecco's Modification of Eagle's Medium supplemented with 10% fetal bovine serum; while 7860 cells were maintained in 1640 medium supplemented with 10% fetal bovine serum. All cell lines, unless otherwise specified, were routinely kept in a 37°C, 5% CO_2_ incubator. UW solution was purchased from Bristol-Myers Squibb (New York, U.S.) for organ preservation.

### Clinical sample collection

Human kidney specimens were obtained from six civilian patients undergoing radical nephrectomy for clear cell renal cell carcinoma (ccRCC) at General Hospital of Jinan Military Command in China. The collection and use of the samples were reviewed and approved by the institutional ethics committees of General Hospital of Jinan Military Command, and all patients provided written informed consent. Radical nephrectomy was conducted by independent surgeons on these patients, all of whom exhibited renal cancers without evidence of macroscopic necrosis upon CT scan and had not undergone other anti-cancer treatments. The surgeons objectively informed patients of the advantages and disadvantages of radical nephrectomy and partial nephrectomy. The surgeons notified the research team to seek patient permission for the use of surgical specimens only after the patient clearly understood the different treatment options and subsequently chose radical nephrectomy. Expedited pathological diagnosis and staging of these specimens was performed prior to sampling and transporting them for research. In total, we obtained six tissue samples diagnosed by pathologists as ccRCC without obvious necrosis. The diameter of these specimens was 3.6 cm, 3.8 cm, 4 cm, 4.3 cm, 4.7 cm, and 4.8 cm, respectively.

Once kidneys were resected, some cancer tissue and matched normal kidney tissue were collected within 30 minutes of warm ischemic time; subsequently, three whole cancerous kidneys were perfused with UW solution, following the manufacturer's instructions, and cold-stored in UW solution, while the other three whole kidneys were perfused with and cold-stored in normal saline as controls. Additional cancer tissue and matched normal kidney tissue were removed after cold-storage of 6 h, 12 h, and 24 h, respectively. Matched normal kidney tissues, located at least 5.0 cm away from visible tumor lesions, were also collected. For each of the above samples, an aliquot was snap-frozen in liquid nitrogen and stored at −80°C for later western blotting analysis, while another aliquot was immersed in normal saline for isolation of primary cell cultures.

### Western blot analysis for Ki7

Proteins in research specimens were extracted as follows. For normal and tumor samples stored at −80°C, cryostat sections (10 µm) were cut and placed in lysis buffer on ice for 15 min. The samples were sonicated for 10 s, and centrifuged for 15 min at 16 000×*g*. Equal amounts of extracted protein, as determined by the Bradford protein assay (Bio-Rad, Hercules, U.S.), were separated by sodium dodecyl sulfate (SDS)-8% polyacrylamide gel electrophoresis and blotted onto Polyvinylidene difluoride membranes (GE healthcare, Little Chalfont, England). Membranes were probed with rabbit Ki67 antibody (Abcam, Cambridge, U.S.). After incubation with peroxidase-coupled secondary antibodies, blots were developed using enhanced chemiluminescence reagent and exposed to X-ray films to detect proteins of interest. Membranes were stripped with Re-Blot Plus (Millipore, Billerica, U.S.), and subsequently reprobed for glyceraldehyde-3-phosphate dehydrogenase (GAPDH) (Abcam, Cambridge, U.S.) as a loading control.

### Measurement of cell proliferation

Cell proliferation was quantified using the Cell Counting Kit-8 (CCK-8) assay, as described below. Cells were plated onto 96-well plates at 3000 cells per well in complete medium and allowed to grow for 24 h under normal culture conditions. At 24 h, the media was removed, and cells were then cultured under various experimental conditions. After 0, 6, 12, and 24 h in different experimental conditions, mitochondrial metabolic activity was quantified by the CCK-8 assay (Dojindo, Mashikimachi, Japan) as per manufacturer's instructions. Briefly, 10 µl of the CCK-8 solution was added per well to the experimental culture media, and cells were further incubated for 24 h. The resulting color was assayed at 450 nM using a microplate absorbance reader (Sunrise, Tecan, Switzerland). Each assay was performed in triplicate.

### Data analysis

Negative and positive controls were routinely incorporated for quality control in all the above tests. All analyses were performed using SPSS 13 software. Repeated-measures analysis of variance (ANOVA) tests were used to compare multiple groups, and *p* values of ≤0.05 were considered statistically significant. Results are expressed as mean ± SE.

## Results

**Figure 1:**
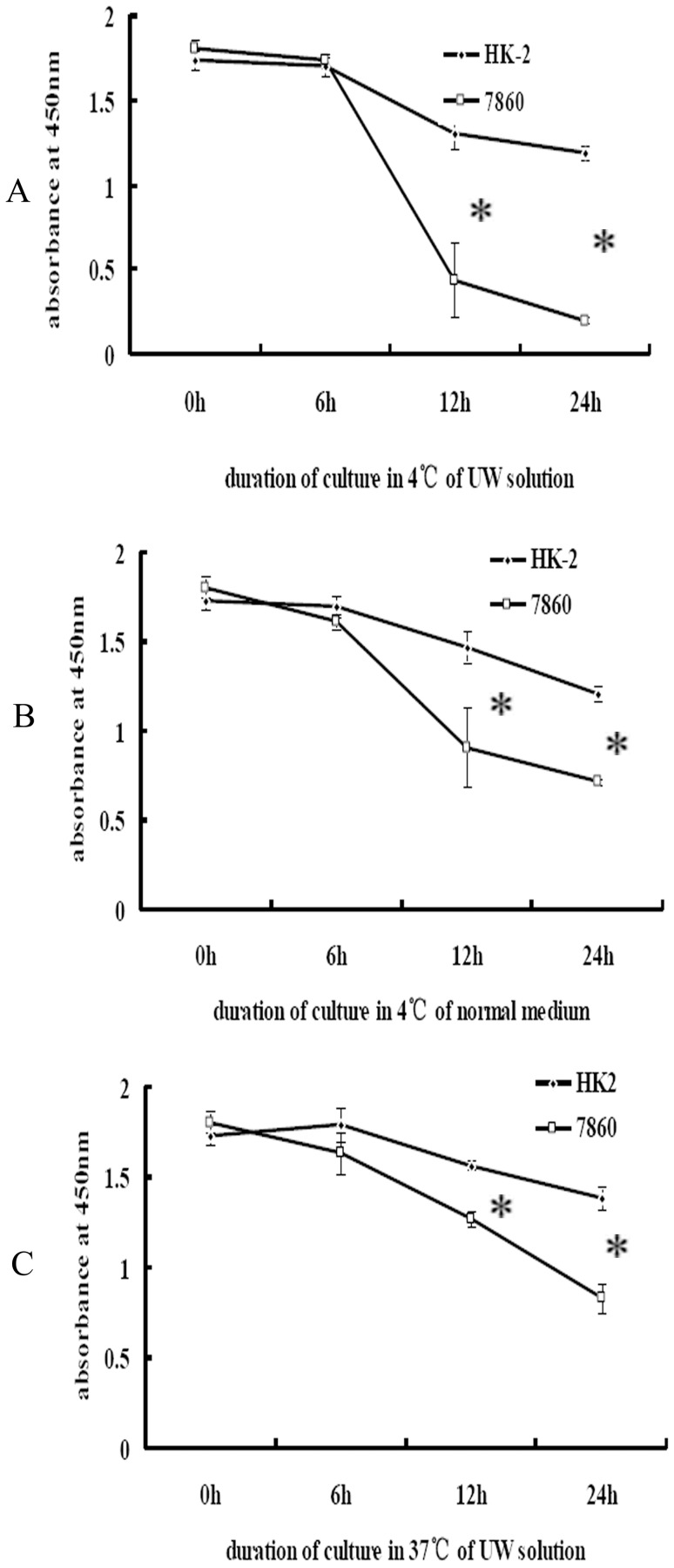
Cellular proliferation under various experimental conditions. Proliferation of HK-2 and 7860 cells after: A, cold-culture (4°C) in UW solution; B, cold-culture in normal medium; and C, culture at 37°C in UW solution. Cells were plated onto 96-well plates at 3000 cells per well in complete medium and cultured for 24 h in a 37°C, 5% CO_2_ incubator. The culture medium was then changed, and wells were divided equally into four groups. Groups 1, 2, 3, and 4 were cold-cultured in UW solution for 0, 6, 12, and 24 h, respectively, as indicated. Subsequently, 10 µl of CCK solution was added to each well, and plates were returned to the incubator for 4 h before the absorbance at 450 nm was measured. *p<0.05.

As shown in Figure 1A, proliferation of both benign (HK-2) and malignant renal cells (7860) was found to be significantly inhibited by cold-storage in UW solution. However, proliferation was inhibited significantly more in malignant renal cells than in benign cells at 12 and 24 h.

**Figure 2:**
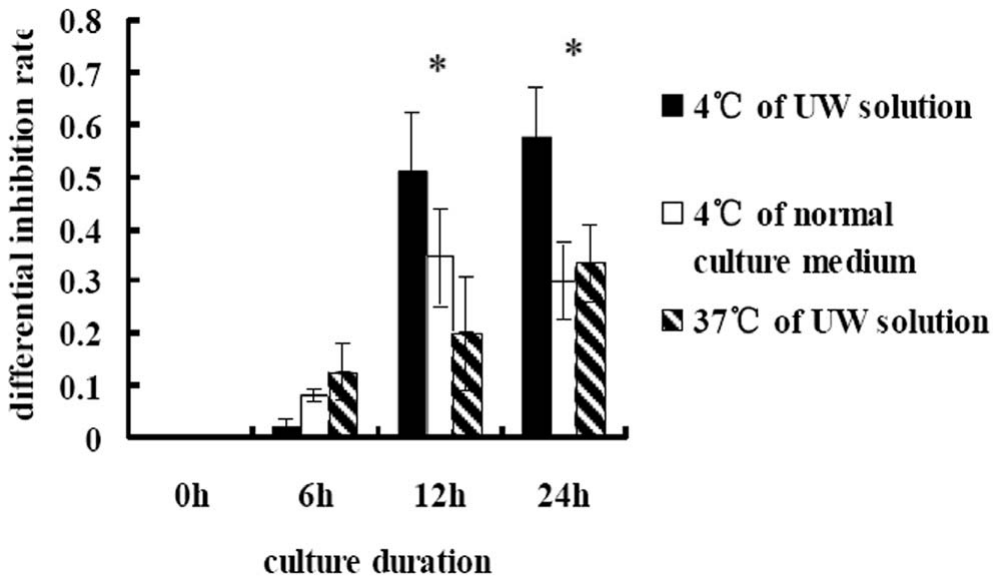
The differential inhibition of proliferation between HK-2 and 7860 cells in differing culture conditions. The differential inhibition rate is the difference between inhibition rate of proliferation of 7860 and HK-2 cells by different culture conditions. *p<0.05.

To further investigate the inhibition of proliferation was induced by hypothermia or by the UW solution, cells were cold-cultured (at 4°C) in normal culture medium or °C in UW solution. As shown in Figure 1B, proliferation of both benign and malignant renal cells was significantly inhibited by hypothermia, though to a significantly greater extent in malignant cells. As shown in Figure 1C, proliferation of benign and malignant cells cultured in UW solution at 37°C was significantly inhibited, and proliferation of malignant renal cells was inhibited significantly more than benign cells. Furthermore, as shown in Figure 2, this differential inhibition between benign and malignant renal cells cold-cultured in normal culture medium, or at 37°C in UW solution, was less than that observed in cells cold-cultured in UW solution.

**Figure 3:**
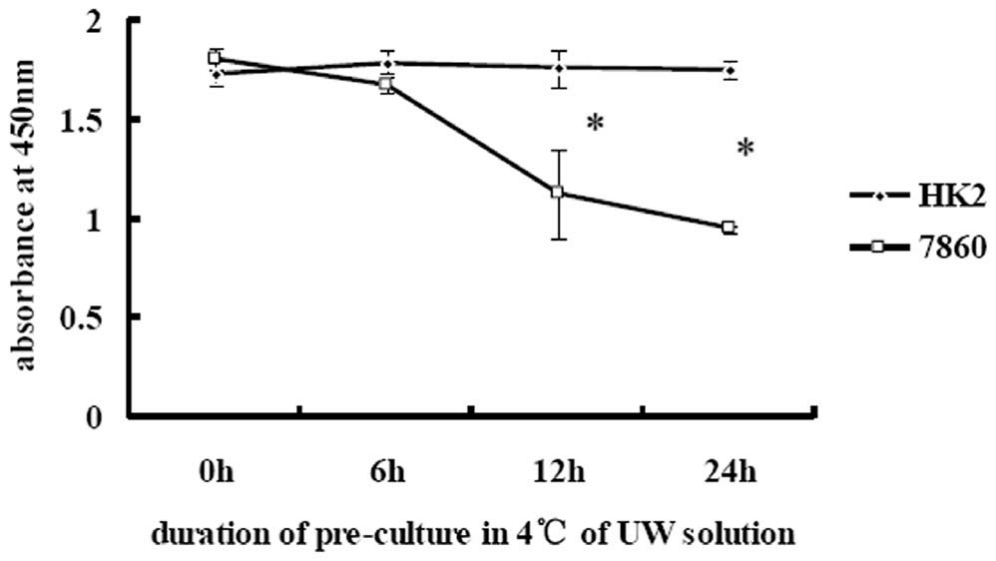
Proliferation of HK-2 and 7860 cells after cold-culture in UW solution and recovery in normal conditions. Cells were cold-cultured in UW solution for the indicated length of time and harvested. Harvested cells were plated onto 96-well plates at 3000 cells per well in complete medium and allowed to grow for 20 h in a 37°C, 5% CO_2_ incubator. Subsequently, 10 µl of CCK solution was added to each well and plates were returned to the incubator for another 4 h before the absorbance at 450 nm was measured. *p<0.05.

To further investigate the reversibility of inhibited proliferation, cells were cold-cultured in UW solution for 6, 12, and 24 h, respectively, and allowed to recover under normal culture conditions for 24 h. Following this, a cell proliferation assay (CCK-8) was performed. Results in Figure 3 indicate that, for benign cells, the inhibition caused by cold-culture in UW solution was reversible; however, the inhibition of proliferation was not completely reversed in malignant cells cold-cultured longer than 12 h in UW solution.

**Figure 4:**
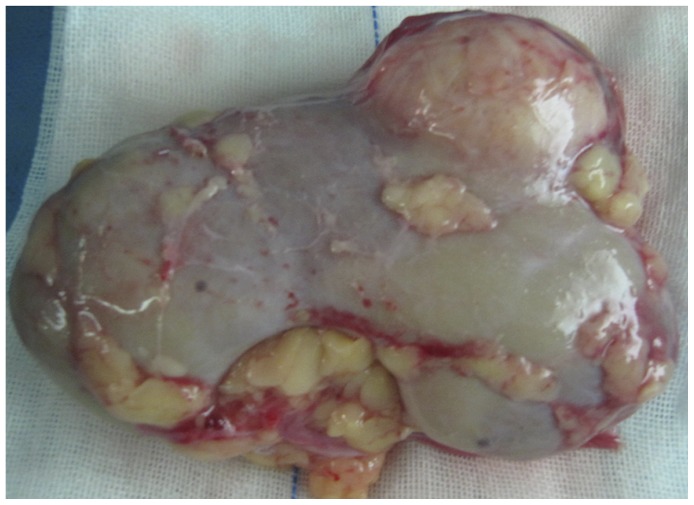
Kidneys with small renal cancer after perfusion with 4°C UW solution. All three renal cancers were less than 4.8(ccCRC).

**Figure 5:**
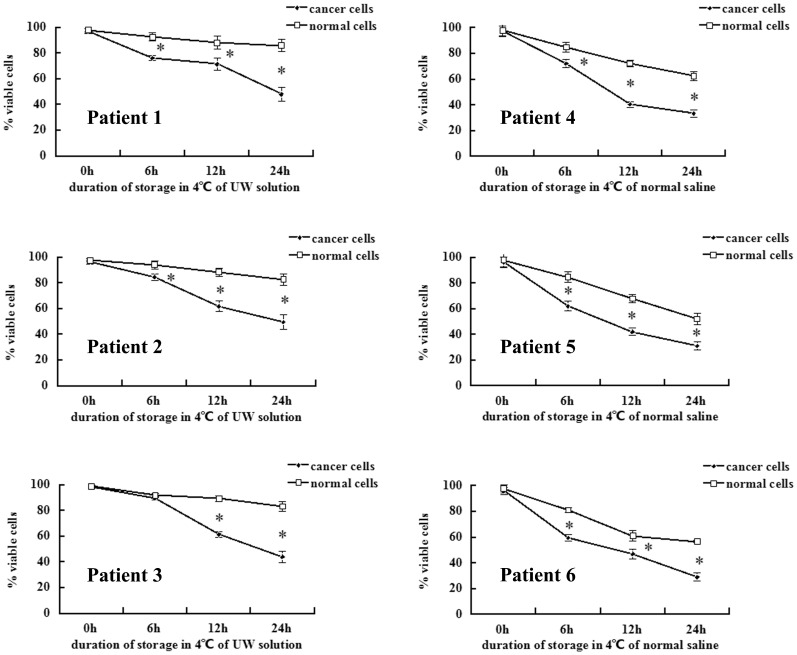
Percentage of viable cells after cold-storage in UW solution or normal saline. After culture for the indicated length of time, viable and dead cells were counted by Trypan Blue exclusion and microscopy. *p<0.05.

Three clinical specimens were perfused and cold-stored in UW solution immediately after resection (Figure 4) or after 6, 12, and 24 h, respectively. Tumor cells and normal cells were isolated, and both viable and dead cells were counted by Trypan Blue exclusion. Significantly fewer viable cancer cells were present in UW solution at 12 and 24 h compared to normal cells (Figure 5, Patients 1, 2, 3.). To investigate whether the differential inhibition caused by cold-culture in UW solution was solution-specific, the remaining three whole kidneys were perfused with and cold-stored in normal saline as control. Cancer cell viability was significantly reduced by cold-storage in normal saline, as compared to normal cells, while the viability of benign and malignant renal cells cold-stored in normal saline was both less than that of cells cultured at 4°C in UW solution (Figure 5, Patients 4, 5, 6.).

**Figure 6:**
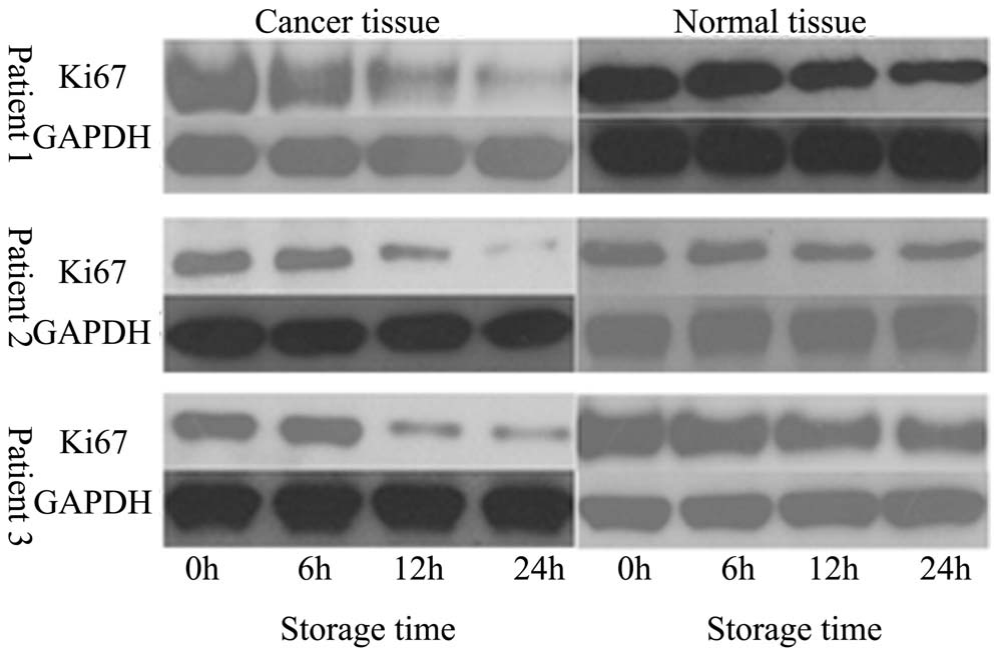
Expression of Ki67 following organ preservation. Protein samples were separated by SDS-PAGE, transferred onto membranes, and probed with anti-Ki67 antibodies. Stripped membranes were re-probed with anti-GAPDH antibodies as a loading control.

To further investigate the above phenomenon on a molecular level, western blot analysis was employed to test the change in Ki67 in renal cancer or normal renal tissue after cold-storage in 4°C of UW solution. As shown in Figure 6, a significant decrease in Ki67 was observed in renal cancer cells cold-stored in UW solution after 6, 12, or 24 h, compared to similarly preserved normal renal cells.

## Discussion

To the best of our knowledge, this is the first study showing that the viability and proliferation of malignant renal cells are inhibited much more than that of benign renal cells during prolonged organ preservation. The inhibition of proliferation in benign renal cells is fully reversible, while in malignant renal cancer cells is not fully reversible after a certain time.

This observation is interesting and creates an opportunity for additional investigations to explore the mechanism of this phenomenon. Though the metabolic rate of cells in cold-storage decreases greatly, energy demand during this and other common organ preservation processes is still higher than the production capacity, which leads to a time-dependent decrease in cellular viability. Cancer cells usually have much higher demands for oxygenation and nutrition, especially those of renal cell cancer, which are strongly associated with neovascularization. Therefore, it is reasonable that renal cancer cells are more vulnerable than normal renal cells under anaerobic and nutrition deprivation conditions created by common organ preservation processes. Control experiments with cold-storage of human kidney tumor in normal saline proved that the differential inhibition of viability observed in benign and malignant renal cells during prolonged organ preservation was not solution-specific, but may result from differential viability of benign and malignant renal cells under conditions of hypothermia and oxygen/nutrient deprivation during common organ preservation processes. Previous studies have suggested that benign and malignant cells respond differentially to hypothermia. Matijasevic [17] reported that the number of viable p53 wild-type cells remained constant, while the number of viable p53-deficient cells declined after several days of hypothermic (28°C) culture conditions. Because wild-type cells respond to hypothermia by arresting cell cycle progression, cellular tolerance to hypothermia may be increased. However, p53-deficient cells did not exhibit hypothermia-induced cell cycle arrest; thus, no such protective effect was observed [17]. Unlike other carcinoma cells, most renal cancer cells are not p53-deficient or -mutant [18]; however, other mechanisms could render the p53 pathway nonfunctional [19]. Therefore, the possibility of p53 dysfunction might also explain the observations in the present study.

To further investigate the mechanism of the differential inhibitory effects of organ preservation, more studies are warranted to determine the nature of cell death from apoptosis, necrosis, or autolysis.

The results of the present study suggest that, after resection, potential residual cancer cells in small renal cancer donor kidneys could be selectively inhibited or partly eliminated by the organ preservation process. This may also explain why current evidence shows a relatively low recurrence rate from transplantation of resected kidney from small renal cancer donors [20]. A systematic literature review revealed 96 documented cases of renal transplants conducted with donor kidneys from small renal cancer patients, with no definite cancer recurrence [21].

Kidneys to be transplanted are usually preserved in solution for several hours in clinical practice, though not for 24 h (as in our experiments), since increased preservation time has been linked with delayed graft function and decreased graft survival [22]. However, the maximum clinically acceptable preservation time for a human kidney is approximately 30–40 h with cold-storage; thus, the 24 h of cold organ preservation in our experiment would be considered acceptable by most transplant units [23]. According to the findings of the present study, a relatively longer preservation time, such as 24 h, might help to decrease the risk of cancer recurrence after allotransplantation of kidneys with small renal cancer.

It has been reported that 33.9% of renal cancers presented with microscopic necrosis [24], while 27% showed macroscopic necrosis [25]. To lessen the avoidable impact of necrosis on our experimental outcome, we excluded renal cancers with macroscopic necrosis by CT scan. Coincidently, the six renal cancer samples used in the present study did not present with microscopic necrosis. Theoretically, the differential inhibitory rate of the organ preservation process on renal cancer cells with necrosis compared to normal kidney cells is unlikely to be lower-if not higher-than that for renal cancer cells without necrosis, since cells prone to necrosis are usually more fragile.

Some limitations exist in the present studies. First, many of the cold-cultured cells were in suspension rather than adherent to culture wells, which may have some impacts on the CCK-8 assay results. Second, cell lines in this study may be not an optimal analytical tool, since the immortalization of cell lines may alter the biology of cells. However, the use of cell lines allowed repeated analysis of genetically identical cells and inspired us to further analyze clinically derived primary cells. Results from our clinical sample study showed the same trend as that in cell lines. Thus, the results from the primary and secondary cell line experiments reinforced each other to allow the current conclusion.

Though cancer cells could be partly inhibited and eliminated by the organ preservation process as indicated by the present study, since not all of the cancer cells could be eliminated, more basic research is warranted to further eliminate potential residual cancer cells before the transplantation of donor kidneys from small renal cancer patients can be widely implemented in clinic. For patients with small renal cancer, current evidence shows that partial nephrectomy, as compared to radical nephrectomy, provides equivalent oncologic outcomes with optimal preservation of renal function; however, a large proportion of patients with small renal cancer choose to undergo radical nephrectomy for various reasons. It is estimated that as many as 8000 to 10 000 cases of small renal cancer are treated by radical nephrectomy in the United States each year [26]. Currently, almost all of these resected kidneys with small renal cancer are discarded. The potential to increase the organ-donor pool with these marginal organs exists, if the potential residual cancer cells can be effectively eliminated.

## Conclusion

By testing in cell lines and clinical samples, we found that the viability and proliferation of malignant renal cells are inhibited much more than that of benign renal cells during prolonged organ preservation. The inhibition of cell proliferation is not completely reversible after a certain time in malignant renal cancer cells, but is fully reversible in benign renal cells.
