# Correction: Benign and Malignant Renal Cells Are Differentially Inhibited during Prolonged Organ Preservation

**DOI:** 10.1371/annotation/b51ed377-9d16-4082-b714-369fc36a8a22

**Published:** 2014-01-14

**Authors:** Nengwang Yu, Shuai Fu, Yubao Liu, Zhihou Fu, Jianzhong Meng, Zhonghua Xu, Baocheng Wang, Aimin Zhang

In the byline, the name of the third author is spelled incorrectly. The correct name is: Yubao Liu.

In addition, a term was spelled incorrectly in the title of the third section of the Materials and Methods. The correct title of the third section is: "Western blot analysis for Ki67".

